# Genome-wide analyses reveal a strong association between LEPR gene variants and body fat reserves in ewes

**DOI:** 10.1186/s12864-022-08636-z

**Published:** 2022-06-01

**Authors:** Tiphaine Macé, Eliel González-García, Didier Foulquié, Fabien Carrière, Julien Pradel, Christian Durand, Sebastien Douls, Charlotte Allain, Sara Parisot, Dominique Hazard

**Affiliations:** 1 GENPHYSE, Université de Toulouse, INRAE, ENVT, 24 chemin de Borde Rouge, CS 52627, F-31326 Castanet-Tolosan, France; 2 SELMET, INRAE, CIRAD, Montpellier SupAgro, Univ. Montpellier, 34060 Montpellier, France; 3 UE321 Domaine de la Fage, INRAE, 12250 Saint-Jean Saint-Paul, France

**Keywords:** Animal genomics, Adaptation, Adipose tissue, Ruminants

## Abstract

**Background:**

Among the adaptive capacities of animals, the management of energetic body reserves (BR) through the BR mobilization and accretion processes (BR dynamics, BRD) has become an increasingly valuable attribute for livestock sustainability, allowing animals to cope with more variable environments. BRD has previously been reported to be heritable in ruminants. In the present work, we conducted genome-wide studies (GWAS) in sheep to determine genetic variants associated with BRD. BR (i.e. levels) and BRD (i.e. changes over time) were obtained through body condition score measurements at eight physiological stages throughout each productive cycle in Romane ewes (*n* = 1034) and were used as phenotypes for GWAS. After quality controls and imputation, 48,593 single nucleotide polymorphisms (SNP) were included in the GWAS.

**Results:**

Among the 23 QTL regions identified, a major QTL associated with BR during pregnancy and lactation was identified on chromosome 1. In this region, several significant SNPs mapped to the leptin receptor gene (LEPR), among which one SNP mapped to the coding sequence. The point mutation induces the p.P1019S substitution in the cytoplasmic domain, close to tyrosine phosphorylation sites. The frequency of the SNP associated with increased BR levels was 32%, and the LEPR genotype explained up to 5% of the variance of the trait. Higher fatness levels in ewes carrying the LEPR p.P1019S mutation were observed all along the productive cycle.

**Conclusions:**

These results provide strong evidences for involvement of LEPR in the regulation of BR in sheep and highlight it as a major candidate gene for improving adaptive capacities by genetic selection.

**Supplementary Information:**

The online version contains supplementary material available at 10.1186/s12864-022-08636-z.

## Background

Breeding farm animals for adaptive traits is of growing interest for the improvement of livestock sustainability. Due to climate change and its associated challenges, it is expected that feed supply fluctuations will increase, both in terms of quantity and quality [1]. To cope with such nutritional challenges, animals rely on their energetic body reserves (BR) present in adipose tissues. Alternation of BR use and accretion periods, referred to as body reserve dynamics (BRD; i.e., lipid mobilization and accretion processes) in the present study, provides animals with a metabolic plasticity that allows them to respond to energetic challenges (for a review, see [2]). Inclusion of BRD in future genetic programs is of particular interest to improve the adaptive capacities of animals and to optimize feeding management, especially for ruminants whose farming systems increasingly rely on rangeland and roughage resources [3].

Some long-term energetic challenges are predictable and result in anticipatory changes in BR, e.g., the high energetic cost for lactation in mammals (for a review see [2]). In this context, the temporal pattern of changes in BR can be genetically-driven. Genetic variability for adiposity has been previously described for several animal species, including humans (for a review, see [4–7]). In ruminant species, total body energy content is a heritable trait in dairy cows throughout lactation [8]. Heritability of BR levels estimated through the body condition score (BCS), a common proxy used to estimate BR in livestock and highly correlated with total body fat content, ranged between 0.08 and 0.45 for dairy cows and sheep [9–11], depending on the breed and the physiological stage of the measurement [12–14]. Heritability of BRD ranged from 0.01 to 0.16 in ruminants [14–17]. These results indicated that BR levels and BRD were heritable traits in ruminants. Such genetic variability in temporal patterns of changes in BR could be used in genetic selection to improve adaptation and resilience while maintaining optimal productivity for a given environment and management system. Recent developments in animal breeding provide the opportunity to include favorable polymorphisms for traits of interest through selection on one or few genotypes. However, to our knowledge, while some studies have reported QTLs in sheep for fatness in the carcasses of lambs [18–21], no such studies have been undertaken for BR phenotypes on live productive females. Given the moderate to high heritability for BR phenotypes in sheep, we hypothesized the existence of some QTL regions underlying BR levels and BRD in productive ewes, among the 26 sheep chromosomes. Therefore, the objective of this study was to detect genetic variants associated with BR levels and BRD traits in ewes. These results should provide new insights to enhance our knowledge of fatness in mammals and molecular data that can be used to improve adaptive capacities in sheep by genomic selection.

## Results

### Descriptive statistics

The BR and BRD were significantly affected by the parity and the age at first lambing of the ewe, the litter size class, and the year of measurements at several physiological stages (Tables 1 and 2, Additional file 1: Table S1, Table S2). The first-order interaction parity × litter size class was significant for BR at all physiological stages and was only significant for BRD between pregnancy and early suckling (BCS-Pa:L, BCS-Pa:W and BCS-L-Sa. Re-ranking of parities across liter size classes was not obversed but the effect of parity was lower with the increase in litter size class. The first-order interaction age at first lambing × litter size class was significant for BR except at lambing, and only significant for BRD between pregnancy and weaning (BCS-Pa:W). Re-ranking of age at first lambing across litter size classes was not observed but the effect of age at first lambing was lower with the increase in litter size class.

**Table 1:** Least-square means for body reserves (± standard error) at each physiological stage of ewes according to parity, litter size class and age of the ewe at first lambing

		% Obs.	BCS-M	BCS-Pa	BCS-Pb	BCS-L	BCS-Sa	BCS-Sb	BCS-W	BCS-Wp
N Obs.			2069	2167	2167	2162	1983	1317	2084	1776
Parity	1	47.3	2.91 (0.01) a	2.96 (0.01) a	2.76 (0.01) a	2.62 (0.01) a	2.52 (0.01) a	2.55 (0.01) a	2.48 (0.008) a	2.57 (0.01) a
	2	38.9	2.69 (0.01) b	2.87 (0.01) b	2.73 (0.01) b	2.65 (0.01) b	2.57 (0.01) b	2.64 (0.02) b	2.56 (0.01) b	2.67 (0.01) b
	3	13.8	2.73 (0.02) c	2.95 (0.02) ac	2.84 (0.02) c	2.70 (0.02) c	2.65 (0.02) c	2.64 (0.02) b	2.61 (0.01) c	2.66 (0.02) b
	Sign.		***	***	***	***	***	***	***	***
Litter size class	0	13.3	2.79 (0.02) ac	2.93 (0.02)	2.79 (0.02) ab	2.75 (0.02) a	2.82 (0.02) a	3.04 (0.03) a	2.97 (0.02) a	2.89 (0.03) a
	1	20.5	2.78 (0.02) ac	2.94 (0.01)	2.83 (0.01) b	2.76 (0.01) a	2.67 (0.01) b	2.65 (0.02) b	2.55 (0.01) b	2.68 (0.01) b
	2	24.3	2.80 (0.01) ab	2.93 (0.01)	2.77 (0.01) a	2.62 (0.01) b	2.54 (0.01) c	2.55 (0.02) c	2.49 (0.01) c	2.58 (0.02) c
	3	24.6	2.78 (0.01) ab	2.93 (0.01)	2.76 (0.01) a	2.63 (0.01) b	2.47 (0.01) d	2.42 (0.02) d	2.39 (0.1) d	2.52 (0.1) d
	4	17.3	2.74 (0.02) c	2.91 (0.02)	2.72 (0.02) c	2.53 (0.01) c	2.40 (0.01) e	2.39 (0.02) e	2.34 (0.01) e	2.48 (0.02) d
	Sign.		*	NS	***	***	***	***	***	***
Age at first lambing	1	47.5	2.83 (0.01) a	2.90 (0.01) a	2.76 (0.01)	2.66 (0.01)	2.58 (0.01)	2.60 (0.02)	2.53 (0.01) a	2.56 (0.01) a
	2	52.5	2.73 (0.01) b	2.96 (0.01) b	2.78 (0.01)	2.66 (0.01)	2.57 (0.01)	2.62 (0.02)	2.57 (0.01) b	2.70 (0.01) b
	Sign.		***	***	NS	NS	NS	NS	***	***
Year	Sign.		***	***	***	***	***	***	***	***
Parity*Litter size class	Sign.		***	*	*	*	***	***	***	***
Age at first lambing*Litter size class	Sign.		***	*	***	NS	*	***	*	**

The significance probabilities were also reported for the year of measurement and first-order interactions

*BCS* Body condition Score, *N Obs* number of records for each trait, % Obs percentage of observations for each class of a given factor, *M* Mating, *Pa* Early pregnancy, *Pb* Two-thirds pregnancy, *L* Lambing, *Sa* Early suckling, *Sb* End of suckling, *W* Weaning, *Wp* Post-weaning period, Sign., the significance probabilities for each fixed effect of mixed models are provided as*:* *** *P*-value < 0.001, ** *P*-value < 0.01, * *P*-value < 0.05; NS, not significant. The lower case letters (a, b, c, d, e) indicate significant differences in the trait between classes of each factor (i.e., values not sharing a common letter are significantly different, as determined by a t-test at *p* < 0.05)

**Table 2:** Least-square means for body reserve dynamics (± standard error) over successive physiological stages of ewes according to parity, litter size class and age of the ewe at first lambing

		% Obs.	BCS-M:Pa	BCS-Pa:L	BCS-Pa:W	BCS-L:Sa	BCS-W:Wp	BCS-W:M
N Obs.			2060	2153	2075	1978	1730	1204
Parity	1	47.3	0.06 (0.01) a	−0.35 (0.01) a	− 0.49 (0.01) a	− 0.10 (0.01) a	0.09 (0.01) a	0.21 (0.01) a
	2	38.9	0.19 (0.01) b	−0.22 (0.01) b	− 0.31 (0.01) b	− 0.08 (0.01) a	0.12 (0.01) b	0.17 (0.02) ab
	3	13.8	0.22 (0.02) b	−0.25 (0.02) b	− 0.32 (0.02) b	− 0.04 (0.02) b	0.08 (0.02) ab	0.12 (0.04) b
	Sign.		***	***	***	**	NS	**
Litter size class	0	13.3	0.12 (0.02) a	−0.18 (0.03) a	0.07 (0.03) a	0.09 (0.03) a	−0.02 (0.03) a	− 0.12 (0.06) a
	1	20.5	0.17 (0.02) ab	−0.17 (0.02) a	− 0.38 (0.02) b	− 0.10 (0.01) bd	0.12 (0.02) bc	0.17 (0.02) b
	2	24.3	0.15 (0.02) ab	− 0.33 (0.02) b	−0.45 (0.02) c	− 0.07 (0.01) b	0.10 (0.02) b	0.23 (0.02) c
	3	24.6	0.16 (0.02) ab	−0.30 (0.01) b	− 0.54 (0.01) d	− 0.16 (0.01) c	0.12 (0.01) bc	0.27 (0.02) cd
	4	17.3	0.18 (0.02) b	−0.37 (0.02) c	− 0.57 (0.02) d	− 0.13 (0.01) cd	0.14 (0.02) c	0.29 (0.02) d
	Sign.		NS	***	***	***	***	***
Age at first lambing	1	47.5	0.08 (0.01) a	−0.23 (0.01) a	− 0.36 (0.01)	− 0.08 (0.01)	0.04 (0.01) a	0.12 (0.02) a
	2	52.5	0.23 (0.01) b	−0.30 (0.01) b	−0.38 (0.01)	− 0.07 (0.01)	0.15 (0.01) b	0.22 (0.02) b
	Sign.		***	***	NS	NS	***	***
Year	Sign.		***	***	***	***	***	***
Parity*Litter size class	Sign.		NS	*	***	*	NS	NS
Age at first lambing*Litter size class	Sign.		NS	NS	***	NS	NS	NS

The significance probabilities were also reported for the year of measurement and first-order interactions

*BCS* Body Condition Score, *N Obs* number of records for each trait, % Obs percentage of observations for each class of a given factor, *M:Pa* Mating to Early pregnancy, *Pa:L* Early pregnancy to Lambing, *Pa:W* Early pregnancy to Weaning, *L:Sa* Lambing to Early suckling, *W:Wp* Weaning to Post-weaning, *W:M* Weaning to Mating. Sign., the significance probabilities for each fixed effect of mixed models are provided as: *** *P*-value < 0.001, ** *P*-value < 0.01, * *P*-value < 0.05; NS, not significant. The lower case letters (a, b, c, d) indicate significant differences in the trait between classes of each factor (i.e., values not sharing a common letter are significantly different as determined by a t-test at *p* < 0.05)

Globally, a significant increase in BR was observed for BCS-Pb, BCS-L, BCS-Sa, BCS-Sb, BCS-W and BCS-Wp with parity, and a decrease in BR was observed for BCS-M and BCS-Pa with parity (Table 1). A significant increase in BR gain was observed for BCS-M:Pa, whereas a decrease in BR gain was observed for BCS-W:M with the increase in parity (Table 2). A significant decrease in BR loss was observed for BCS-Pa:L, BCS-Pa:W and BCS-L:Sa with the increase in parity. The litter size class effect was significant for BCS-M, BCS-Pb, BCS-L, BCS-Sa, BCS-Sb, BCS-W and BCS-Wp with a decrease in BR for larger litter size (Table 1). BR loss increased for BCS-Pa:L, BCS-Pa:W, BCS-L:Sa and BR gain increased for BCS-W:Wp and BCS-W:M with the increase in litter size (Table 2). The age at first lambing was significant for BR, except between two-thirds pregnancy and end of suckling, with a higher BR for younger ewes at BCS-M and a lower BR for younger ewes at BCS-Pa, BCS-W and BCS-Wp (Table 1). A significant increase in BR gain was observed for BCS-M:Pa, BCS-W:Wp and BCS-W:M, and a significant increase in BR loss was observed for BCS-Pa:L for ewes that lambed at 2 years of age (Table 2). The year effect was significant (*P* < 0.01) for BR and BRD at all physiological stages.

### Genome-wide association studies

The QTLs reaching the chromosome-wide (CW) or genome-wide (GW) thresholds are reported in Table 3. Six QTLs reached the GW significance threshold and 17 reached the CW significance thresholds. Among these 23 QTLs, 16 were related to BR and seven were related to BRD. These QTLs were located on 12 different chromosomes. Estimated QTL effects ranged from 1.6 (BCS-W:Wp on OAR25) to 3.9% (BCS-L on OAR1) of the respective phenotypic variance.

**Table 3:** Summary of QTLs detected in GWAS and candidate genes associated with body reserves and body reserves dynamics

Trait^a^	Chr	Nb SNPs^b^	Top SNP^c^	Position (bp)	-log10(*P*-value)	SNP effect^d^	Closest gene^e^
**BCS-Pb**	**1**	**3**	**OAR1_40030112.1**	**38,809,812**	**6.55**	**3.7**	***PGM1***
BCS-L	1	1	OAR1_40030112.1	38,809,812	5.87	3.3	*PGM1*
BCS-Pa	1	2	s30054.1	40,524,081	5.60	2.7	*DNAJC6*
**BCS-Sa**	**1**	**3**	**oar3_OAR1_40821987**	**40,821,987**	**6.06**	**2.8**	***LEPR***
**BCS-Pb**	**1**	**7**	**oar3_OAR1_40,857,869**	**40,857,869**	**6.90**	**3.5**	***LEPR***
**BCS-L**	**1**	**11**	**oar3_OAR1_40890859**	**40,890,859**	**7.44**	**3.9**	***LEPR***
**BCS-Pb**	**1**	**2**	**OAR1_42228129.1**	**42,228,129**	**6.03**	**2.9**	***MIER1***
BCS-Sb	2	1	OAR2_204233702.1	192,716,355	5.51	2.5	*MYO1B, STAT1*
BCS-Pa:W	3	1	OAR3_115100887.1	115,100,887	5.24	2.4	*SYT1*
BCS-Pb	8	1	s48527.1	13,120,875	4.84	2.0	*TPD52L1*
BCS-Pa	10	1	OAR10_9706403.1	11,281,919	5.00	2.0	*PCDH8*
BCS-Pa	15	1	OAR15_26097184.1	24,911,875	4.82	2.3	*NXPE4*
BCS-Sa	15	1	OAR15_87285774.1	78,544,042	5.22	2.3	*OR10W1*
BCS-Sa	15	1	OAR15_87912118.1	87,912,118	4.65	2.2	*–*
**BCS-W:Wp**	**16**	**2**	**OAR16_33763548.1**	**31,368,878**	**6.59**	**2.6**	***CCL28***
BCS-W:Wp	16	1	OAR16_34857607.1	34,857,607	4.99	1.9	*DAB2*
BCS-Pa:L	16	1	OAR16_46544413.1	42,819,279	5.03	2.0	*CDH6*
BCS-Sa	17	1	s70069.1	14,052,727	4.47	2.3	*FREM3, GAB1*
BCS-L	18	1	OAR18_31578626.1	30,304,217	4.65	2.4	*NRG4*
BCS-M:Pa	22	1	OAR22_24239807.1	24,239,807	4.39	1.9	*SORCS3*
BCS-Pa:L	24	1	OAR24_22245800.1	20,494,588	4.20	1.9	*HS3ST2*
BCS-W:Wp	25	1	s68395.1	32,693,219	4.43	1.6	*KCNMA1*
BCS-Pa	25	1	OAR25_39067458.1	37,133,591	4.75	2.5	*–*

^a^ BCS, Body Condition Score; M, Mating; Pa, Early pregnancy; Pb, Two-thirds pregnancy; L, Lambing; Sa, Early suckling; Sb, End of suckling; W, Weaning; Wp, Post-weaning period

^b^Number of significant SNPs in the 1-Mb window

^c^The reported top SNPs are SNPs that have the highest –log10 (*P*-value) among the significant SNPs that are in 1-Mb windows. SNPs for which –log10 (*P*-value) reached the genome-wide significance threshold (> 5.98) are reported in bold; the other reported SNPs reached the chromosome-wide significance threshold

^d^Percentage of variance explained by SNP

^e^Annotated protein coding genes closest to the top SNP of the QTL region. Chr, chromosome

**Fig. 1:**
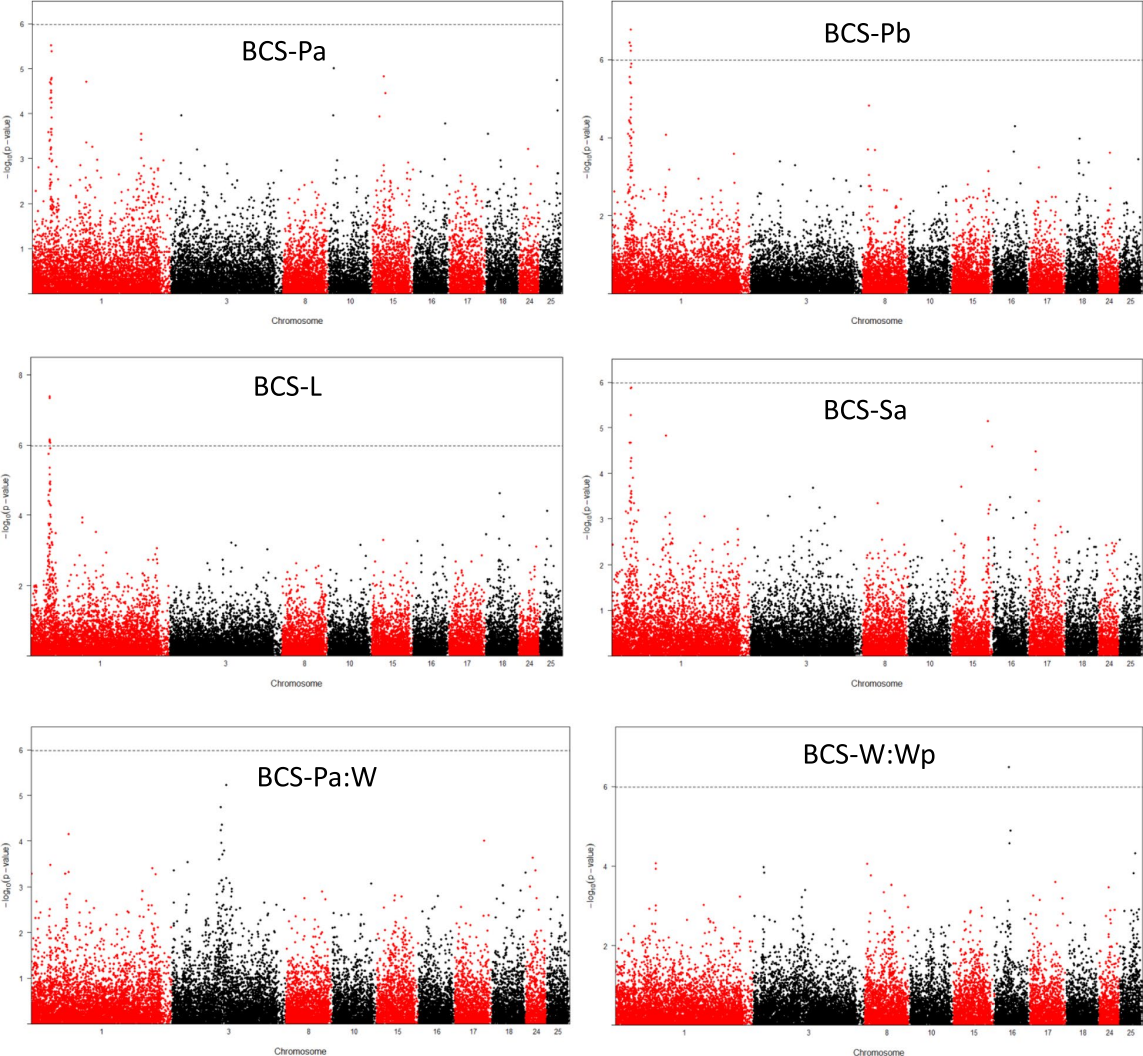
Chromosome plots of body reserves and body reserves dynamics. The –log_10_ (*p*-value) for all SNPs were plotted for chromosomes 1, 3, 8, 10, 15, 16, 17, 18, 24 and 25. The dashed line indicates the genome-wide significance threshold (BONF_gen_ = 5.94); The chromosome-wide significance thresholds were OAR1: 5.02, OAR3: 4.96, OAR8: 4.57, OAR10: 4.52, OAR15: 4.49, OAR16: 4.45, OAR17: 4.42, OAR18: 4.43, OAR24: 4.14, OAR25: 4.26. BCS, Body Condition Score; Pa, Early pregnancy; Pb, Two-thirds pregnancy; L, Lambing; Sa, Early suckling; W, Weaning; Wp, post-weaning

Five of the six significant associations reached the GW significance threshold mapped on OAR1 and were associated with BR (Table 3, Fig. 1). Two additional QTLs reached the CW threshold localized on OAR1. All the significant SNPs mapped on OAR1 were located between 38.80 and 42.22 Mb. A first QTL region mapped on OAR1 at 38.80 Mb was associated with BCS-Pb and BCS-L. A second QTL region on OAR1 located between 40.26 and 41.56 was associated with BR at four physiological stages (BCS-Pa, BCS-Pb, BCS-L and BCS-Sa). A third QTL region located on OAR1 at 42.22 Mb was associated with BCS-Pb. Details of the second QTL region located on OAR1 are given in Table 4. This QTL region contained 14 SNPs, significantly associated with BR at least at one physiological stage. Among these 14 SNPs, four SNPs mapped in the LEPR gene, including three SNPs localized in the intronic sequence and one SNP in the coding sequence.

**Table 4:** Details of the 14 single nucleotide polymorphisms (SNPs) detected in the common OAR1 region associated with body reserves at four physiological stages

SNP	Position	MAF	***P***-value				Gene	Variant type
			BCS-Pa	BCS-Pb	BCS-L	BCS-Sa		
OAR1_41661218.1	40,268,642	0.39	*3.05E-05*	*1.91E-05*	**7.40E-07**	*5.71E-04*	no	intergenic
s30054.1	40,524,081	0.09	2.51E-06	*3.28E-04*	*2.75E-03*	*3.21E-04*	no	intergenic
OAR1_42038601.1	40,539,479	0.36	*2.72E-05*	*3.06E-05*	4.58E-06	1.41E-02	no	intergenic
oar3_OAR1_40821987	40,821,987	0.35	*3.42E-05*	2.74E-06	*1.88E-05*	**8.63E-07**	LEPR	intron
oar3_OAR1_40828247	40,828,247	0.44	*1.55E-05*	**3.61E-07**	**6.81E-07**	3.50E-06	LEPR	intron
oar3_OAR1_40848298	40,848,298	0.32	*2.72E-05*	**1.27E-07**	**4.11E-08**	*4.32E-05*	LEPR	intron
oar3_OAR1_40,857,869	40,857,869	0.32	*2.72E-05*	**1.27E-07**	**4.11E-08**	*4.32E-05*	LEPR	missence
oar3_OAR1_40865586	40,865,586	0.32	*2.72E-05*	**1.27E-07**	**4.11E-08**	*4.32E-05*	no	intergenic
oar3_OAR1_40872161	40,872,161	0.50	*1.10E-02*	*4.60E-04*	6.76E-06	*3.83E-04*	no	intergenic
oar3_OAR1_40890859	40,890,859	0.32	*6.15E-05*	**3.31E-07**	**3.61E-08**	*6.09E-05*	no	intergenic
oar3_OAR1_41137040	41,137,040	0.31	*1.33E-05*	1.17E-06	**6.10E-07**	*2.50E-04*	no	intergenic
OAR1_43022391.1	41,454,757	0.28	*2.61E-04*	*8.85E-05*	1.04E-06	*2.55E-04*	no	intergenic
OAR1_43083702.1	41,512,082	0.62	*1.05E-03*	*1.41E-04*	*1.02E-05*	1.15E-06	no	intergenic
oar3_OAR1_41564208	41,564,208	0.61	3.34E-06	*8.10E-05*	**7.10E-07**	*3.77E-04*	no	intergenic

*SNP* Name of the single nucleotide polymorphism, *MAF* minor allele frequency, *BCS* Body Condition Score; *P*-value, the *p*-values reported correspond to the unadjusted *p*-value from the Wald test. In bold, SNP significant at the genome-wide threshold; in italics, non-significant SNPs at the chromosome-wide threshold. Gene, gene is reported when SNP overlapped with a gene. LEPR, Leptin receptor

One additional significant association was also detected with BCS-Pb and BCS-L and mapped on OAR8 and OAR18, respectively (Table 3). Only one QTL was associated with BCS-Sb and localized on OAR2. Three additional QTLs were associated with BCS-Pa and mapped on OAR10, OAR15 and OAR25 (Table 3). Similarly, three additional QTLs were associated with BCS-Sa and mapped on OAR15 at 78.54 and 87.91 Mb and OAR17. Three QTLs were significantly associated with BCS-W:Wp and mapped on OAR16 at 31.36 and 34.87 Mb and on OAR25. The QTL associated with BCS-W:Wp located on OAR16 at 31.36 Mb reached the GW threshold (Table 3, Fig. 1). Two QTLs were associated with BCS-Pa:L and localized on OAR16 at 42.81 Mb and OAR24. Finally, one QTL was associated with BCS-M:Pa and mapped on OAR22, and one QTL was associated with BCS-Pa:W and mapped on OAR3.

### Leptin receptor structure

**Fig. 2:**
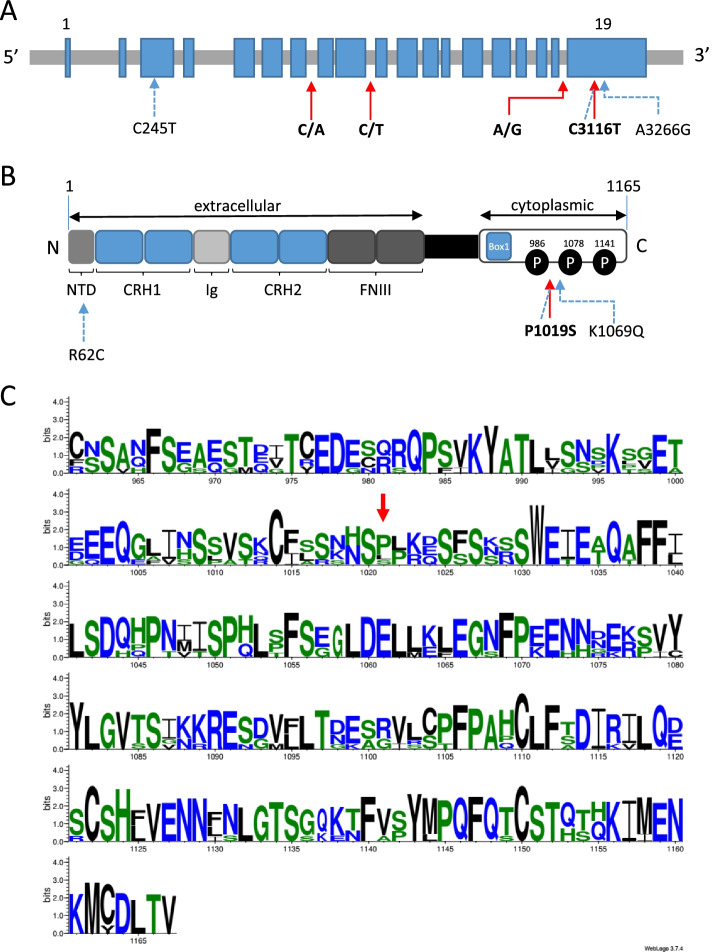
The leptin receptor (LEPR) gene and protein structure and polymorphisms in sheep. **A** Schematic nucleotide sequence structure of the LEPR gene and sheep polymorphisms previously reported to be associated with reproductive phenotypes (dashed arrows) or identified in the present study (solid arrows). Arrows indicate the position within the gene and the type of base pair exchange. Numbers, when reported, indicate position within the cDNA sequence ENSOART00000011314.1. **B** LEPR protein structure based on the long form (1165 amino acids) and polymorphisms previously reported (dashed arrows) or identified in the present study (solid arrow). Arrows indicate the position within the protein and the type of amino acid exchange. NTD: N-terminal domain; CRH: cytokine receptor homology; Ig: immunoglobulin-like domain; FNIII: fibronectin type III; Leptin binds to its homodimer receptor through CRH2 and activates downstream effectors through Box 1 domain and phosphorylation (P) of Y-residues (Y986, Y1078, Y1141) (adapted from Berger et al. [22]). **C** Multiple alignment of the LEPR protein sequences from mouse (NP_666258.2), rat (NP_036728.1), human (NP_002294.2), pig (NP_001019758.1), cattle (NP_001012285.2) and sheep (W5PL31) species with Weblogo software [23]. Only the C-terminal end of the protein is represented. A red arrow indicates the P1019 position in sheep. Numbering on the X-axis resulted from the multiple alignment and not the ovine sequence

Among SNPs significantly associated with BR and located in the LEPR gene, only a single SNP (i.e., oar3_OAR1_40,857,869) mapped to the coding region of the gene (Fig. 2A). The mutation induced a non-synonymous change in amino acid. Modification of the C base in the reference sequence (OAR1, 40,857,869 bp) to a T encoded a proline to serine substitution at position 1019 (p.P1019S) of the leptin receptor protein (Fig. 2B). The mutation is located within the cytoplasmic domain and replacement of proline by serine causes a polar to non-polar amino acid substitution. While tyrosine phosphorylation sites of the cytoplasmic domain were highly conserved across species (i.e., Y986, Y1078, Y1141), this sequence of the LEPR protein was poorly conserved across species in the region surrounding the mutation (Fig. 2C).

### Effects of the LEPR genotype

**Fig. 3:**
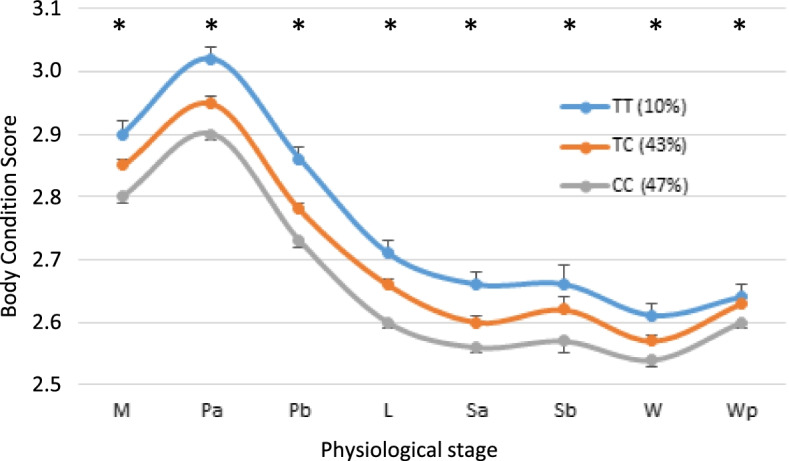
Effect of LEPR genotype on body reserves throughout the productive cycle of ewes. Values represent the lsmeans for body condition score and were obtained from the mixed model with repeated measurements over three successive productive cycles. The asterisk shows the significant overall effect of the LEPR genotype on the trait at *p* < 0.05. The percentage of ewes for each genotype is given between parentheses. M, Mating; Pa, Early pregnancy; Pb, Two-thirds pregnancy; L, Lambing; Sa, Early suckling; Sb, Mid suckling; W, Weaning; Wp, post-weaning

The frequencies of wild-type (C/C), heterozygous (C/T) and homozygous (T/T) carriers were 47, 43 and 10%, respectively. Analysis of variance confirmed that the T mutation involved a significant increase in BR at all physiological stages of the productive cycle, as shown by the average BCS values in each genotype (Fig. 3). Wild-type (C/C) ewes showed the lowest BR all along the productive cycle, whereas heterozygous (C/T) ewes showed intermediary BR levels and homozygous (T/T) ewes exhibited the highest BR. In addition to BCS, the LEPR genotype had significant effects on the body weight, back fat and muscle thickness depending of the physiological stages (Table 5). The homozygous (T/T) ewes had significantly higher body weight than wild-type (C/C) ewes from mating to lambing. The homozygous (T/T) ewes had significantly higher back fat thickness than wild-type (C/C) ewes at mating and end of pregnancy. The homozygous (T/T) ewes had significantly higher back fat muscle than wild-type (C/C) ewes at the last third of pregnancy.

**Table 5:** Effect of the LEPR genotype on body weight (BW), back fat depth (BF) and back muscle depth (BM) of ewes throughout the productive cycle

	TT	TC	CC	Sign.
n	107	442	485	
BW-M	53.20 (0.47) a	52.4 (0.26) ab	51.9 (0.25) b	*
BW-Pa	56.50 (0.5) a	55.4 (0.28) ab	54.86 (0.27) b	*
BW-Pb	61.10 (0.52) a	60.4 (0.29) a	59.6 (0.28) b	*
BW-L	59.5 (0.75) a	58.3 (0.57) ab	57.8 (0.56) b	*
BW-Sa	58.6 (0.58)	58.2 (0.34)	57.8 (0.33)	NS
BW-Sb	60.2 (0.73)	59.8 (0.45)	59.6 (0.44)	NS
BW-W	56.2 (0.55)	56.0 (0.30)	55.7 (0.29)	NS
BW-Wp	57.5 (0.54)	57.3 (0.31)	56.8 (0.30)	NS
BF-M	5.44 (0.3) a	5.1 (0.2) ab	4.9 (0.2) b	*
BF-Pb	5.0 (0.2) a	4.3 (0.2) b	4.2 (0.2) b	**
BF-W	4.5 (0.3)	4.3 (0.2)	4.3 (0.2)	NS
BM-M	22.5 (0.7)	22.3 (0.5)	22.3 (0.5)	NS
BM-Pb	21.5 (0.7) a	19.7 (0.5) b	19.8 (0.5) b	*
BM-W	19.6 (0.9)	19.4 (0.7)	18.9 (0.7)	NS

Values reported are lsmeans (± standard error) obtained from the mixed model with repeated measurements over successive productive cycles. Sign. indicates the significant overall effect of the LEPR genotype on the trait at * *p* < 0.05, ** *p* < 0.01. NS, not significant. Different letter superscripts (a, b, c) show a significant difference between genotypes at *p* < 0.05. n (%), proportion of ewes in each genotype; *M* Mating, *Pa* Early pregnancy, *Pb* Two-thirds pregnancy, *L* Lambing, *Sa* Early suckling, *Sb* End of suckling, *W* Weaning, *Wp* Post-weaning period, *BW* body weight in kg, *BF* back fat thickness in mm, *BM* back muscle thickness in mm

## Discussion

Considering the existing genetic variability for energetic body reserve traits previously reported in sheep [8, 13, 14], the aim of the present study was to provide the first characterization of the genetic architecture that controls body condition in productive ewes. This was achieved by a genome-wide association study (GWAS) of a set of body reserve (BR) traits measured at key physiological stages of the productive cycle in Romane ewes.

### Body reserve dynamics

Body reserve changes throughout ewe productive cycles found for the sheep population used in the present study have been described and discussed in detail by Macé et al. [14, 24]. The BCS is considered as a usual indicator used to describe BR and BRD. Nevertheless, there is a consensus about the subjectivity of BCS that highlight importance of consistent intra- and inter-operator assessment that must be checked regularly. Sources of variation affecting BR and found in the present study were fully consistent with those previously described in our experimental conditions, even if the number of sheep was slightly different since we kept only phenotyped and genotyped ewes. Briefly, body reserve changes over time were highly influenced by physiological stages. Generally, BR accretion was observed from weaning to early pregnancy, whereas BR mobilization was observed from two-thirds pregnancy to weaning, which is probably linked to the negative energy balance induced by the increase in energetic requirements during pregnancy and suckling periods. The BR levels and BR changes over time reported in the present study were also affected by the biological effects of parity, litter size and age of ewes at first lambing. The increase in BR mobilization and accretion with litter size were consistent with the higher energy requirements induced by multiple litters and the high genetic correlation between BR mobilization and accretion processes previously reported by Macé et al. [14]. Interestingly, the increase in BR accretion and the decrease in BR mobilization with parity suggested that ewes, thanks to their metabolic experience in the previous cycles, may develop strategies either linked with BR management or feeding to limit negative effects of excessive BR mobilization. Concerning the year effect on BR and BRD, variation between years were probably mainly due to changes in environmental conditions. Since feed supplementation was limited to the winter period, variations in quality and quantity of the grass in rangelands directly affected BR and BRD.

### QTLs for BR traits

As far as we know, this is the first study on ruminants that maps QTLs for BR levels at several key physiological stages and BR changes over time in ewes. Indeed, many QTLs have been previously found for conformation traits and carcass fatness in sheep [18, 20, 21, 25], but not yet for BR traits in live productive ewes. In the present study, we were not only interested in BR levels assessed through body condition score (BCS) but also in body reserve dynamics (BRD) assessed through BCS changes over time (i.e., between physiological stages). The GWAS analyses resulted in the mapping of many QTLs associated with BR levels (OAR1, 2, 8, 10, 15, 17, 18, 25) and BR changes (OAR3, 16, 22, 24, 25).

The findings were of particular interest for associations mapped on chromosome 1 (OAR1). The QTL regions detected on OAR1 were both associated with several correlated traits and showed a high level of significance. These QTL regions on OAR1 were associated with body reserve levels at pregnancy, lambing and early suckling. Interestingly, we previously reported that BR mobilization occurred between two-thirds pregnancy and weaning in ewes reared in the extensive conditions of the La Fage farm [24]. Thus, the QTLs on OAR1 may be associated with BR levels during the BR mobilization process. Overlapping on OAR1 for QTLs associated with BR levels at several physiological stages of the mobilization period was consistent with the high genetic correlations previously reported for BR levels between physiological stages [14].

The common QTL region on chromosome 1 (40.26 to 41.56 Mb) associated with BR levels at four key physiological stages, harbored several highly significant SNPs, including four SNPs overlapped with the most interesting candidate gene: *LEPR* (leptin receptor). The *LEPR* gene codes for the receptor of the leptin hormone. The leptin hormone, secreted by adipose tissue, and the leptin receptor have been widely described for their major role in energy regulation [4, 5, 22]. Mutations in leptin and LEPR genes have been reported to cause obesity in human and animal models [22, 26–29]. In sheep, by using a candidate gene approach, Haldar et al. [30] described three mutations in LEPR associated first with reproductive traits. One of these mutations found in the Davisdale sheep breed is located at 40,857,869 bp on chromosome 1 and corresponds to the SNP oar3_OAR1_40,857,869 significantly associated with BR levels in the present study. This mutation in the coding region of the gene, modifying a cytosine to a thymidine, causes an amino acid change in the cytoplasmic domain, implying a substitution from proline to serine (p.P1019S), which is not observed in other species. This variation causes a polar to a non-polar amino acid substitution and may alter a potential phosphorylation site from the serine directly preceding it, as suggested by Haldar et al. [30]. Whether this would alter the receptor function remains unknown because the potential role of phosphorylation of serine at amino acid 1018, which is highly conserved across species, has not been examined to date. We also cannot exclude the possibility that additional polymorphisms may exist in the LEPR gene in the Romane sheep breed, which could alter LEPR functional activity.

Many studies have documented the essential role of the LEPR protein in energy regulation. Various mutations in the LEPR gene leading to leptin signaling deficiency through disruption in the LEPR function resulted in many cases in obesity/diabetes phenotypes in humans and rodents [22, 26–29]. In pigs, a missense mutation in the *LEPR* gene was also associated with higher fatness levels [31, 32] and antagonistic maternal and direct effects on body weight [33]. In sheep, in addition to the effect on reproductive traits, Haldar et al. [30] also found an effect of the LEPR p.P1019S mutation on adult body weight, with ewes with the mutation being heavier than the other ewes. They also reported that for ewes homozygous for the LEPR p.P1019S mutation, BR at 18 months of age was 5 to 10% greater than for the wild-type ewes. This is in accordance with our present results showing higher body weight and fatness levels in ewes carrying the LEPR p.P1019S mutation. When using BCS as proxy of fatness, higher fatness levels in ewes carrying the LEPR p.P1019S mutation was observed all along the productive cycle and not only at the key physiological stages of BR mobilization. The range of differences in BR between genotypes was in agreement with the individual variability that we previously reported in our farming conditions [24]. One must keep in mind that the low number of homozygous (T/T) carriers compared to the number in the two other genotypes may have reduced the statistical power of comparison between genotypes and reduced the robustness to unequal variances. There are multiple underlying causes of obesity phenotypes in LEPR mutants, including hyperphagia, increased lipogenesis, or increased feed efficiency [28]. Considering the effects of the LEPR p.P1019S mutation observed in the present study, it may be hypothesized that the present mutation may result in a leptin signaling deficiency in sheep and increased lipogenesis.

Two additional QTL regions were found on OAR1, flanking the QTL region containing the LEPR gene described above, and both are associated with BR levels during pregnancy. Interestingly, one of these QTL region maps close to the gene encoding PGM1 (phosphoglucomutase 1). The protein *PGM1* is known to have a central role in gluconeogenesis and glycolysis in humans [34]. Involvement of PGM1 in energetic metabolism and the QTL found close to this gene in the present study makes PGM1 an additional potential candidate gene for BR regulation in sheep.

Nine additional QTL regions were associated with BR levels but only a single SNP reached the chromosome-wide significance level for each of these regions. These QTLs were mainly associated with BR levels during the BR mobilization period, and only QTLs associated with BR at early pregnancy were found for the BR accretion period. Indeed, no QTLs were found for BR levels post-weaning and at mating. Concerning BR changes over time, only a few QTLs were found. The low number of QTLs associated with BR changes over time could be due to a lower genetic variability for these traits compared to BR levels showing higher genetic variability [14]. Among the four QTLs associated with BR gain, only one QTL region reached the genome-wide significance threshold (OAR 16). Several coding genes are located close to the fine location of the QTL in these regions, although no scientific evidence has yet suggested their involvement in regulating energy balance and/or body fatness. Among these genes, the gene DAB2 codes for a protein that acts as a regulator of the activity of protein serine/threonine kinase and could be involved in the leptin signaling pathway.

## Conclusions

The work reported here is the first SNP-based QTL detection for body reserve traits at key physiological stages in productive ewes. We reported various QTLs, including a major QTL on OAR1 associated with BR levels during the BR mobilization period. This QTL region on OAR1 harbors an interesting candidate gene, LEPR, previously described as being associated with obesity and energy regulation in several species. The present identification of a candidate mutation in the LEPR gene provides new opportunities for a deeper understanding of the genetic regulation involved in body reserve management in mammals. Further studies will be developed to investigate functional consequences on the LEPR protein of the identified mutation and to search for potential additional genetic variants in this gene. The impact of this mutation on production traits will also be investigated before considering this genetic variant in small ruminant breeding schemes in order to improve adaptation.

## Materials and methods

### Animals and management

The experimental animals were Romane ewes reared at the INRAE *La Fage* Experimental Farm (*Causse du Larzac*, Saint-Jean Saint-Paul, southern France) between 2006 and 2019 (*n* = 1034) [35]. Ewes were reared exclusively outdoors on approximately 280 ha of rangelands, in a flock of 250 reproductive females present each year. The main management features of this farming system have been previously described in detail [36–38]. Briefly, the farming system was based on a productive flock reared exclusively in extensive harsh conditions while limiting supplementation, in order to investigate the capacity of ewes to fend for themselves. In the autumn, before mating began, dry ewes successively grazed native and fertilized rangelands (6% of the total surface). A single mating period took place at the end of the autumn and first mating occurred at 8 or 20 months of age depending on the ewe’s live weight and experiment. During the winter and the second half of pregnancy, ewes were gradually supplemented with conserved feedstuffs (i.e., hay and silage produced on the farm) and barley due to the absence of grazeable biomass on the rangelands (for the detailed composition of the diet, see [37]). Lambing took place outdoors in the spring (April) and ewes suckled lambs for approximately 80 ± 4 days while they successively grazed fertilized and native rangelands. During the summer, dry ewes grazed the senescent vegetation due to drought on large paddocks containing a high proportion of shrubs (up to 30%). The Romane ewes produced an average of 2.2 live lambs per lambing in our conditions.

### Measurements

All the ewes were individually monitored for their body reserves (BR) through body condition score (BCS), body weight (BW), subcutaneous back fat (BF) and muscle (BM) depth, and their pedigree information was recorded. All the data were recorded in an INRAE experimental database for sheep and goats (GEEDOC). BCS measurements were performed according the original scale described by Russel et al. [9] (i.e., ranging from 1, emaciated, to 5, obese) and the scale was adapted with a subdivision of 0.1 increments instead of 0.25. The same two operators systematically recorded the BCS measurements over the 14-year period and underwent regular training sessions for calibration. Subcutaneous back fat and muscle depths were measured by a real-time ultrasound system on the 12th rib. The two technicians mentioned above were also involved in BF and BM measurements (performed over the last 4 years of the period) and underwent regular training. The measurements used in this study were collected on a regular basis during one to three productive cycles, according to the following physiological stage schedule: mating (M, 15 days before mating), early pregnancy (Pa, 39 ± 11 days after mating), two-thirds of pregnancy (Pb, 101 ± 11 days after mating), lambing (L), early suckling (Sa, 17 ± 10 days after lambing), middle of the suckling period (Sb, 42 ± 10 days after lambing), weaning (−W, 80 ± 10 days after lambing) and post-weaning period (Wp, 149 ± 11 days after lambing). To characterize body reserves dynamics (BRD), differences in BCS between pairs of physiological stages were calculated and analyzed (BCS-Pa:L, BCS-L:Sa, BCS-Pa:W, BCS-M:Pa, BCS-W:Wp and BCS-W:M; described by Macé et al. [24]). In addition to BCS, BW was also measured at all the stages described above, and BF and BM were measured at mating, lambing and weaning.

### Descriptive statistics

Analyses of variance were carried out, taking the repeated measurements into account, using the MIXED procedure of the Statistical Analysis System (SAS version 9.4; SAS Institute Inc., Cary, NC, USA) to test relevant effects and interactions affecting phenotypes. The age at first lambing, the parity of the ewe, the litter size class and the year of measurement were identified as fixed effects and a random animal effect was included to consider repeated measurements on the same animal. The age at first lambing effect took account of ewes that lambed for the first time at 1 or 2 years of age (classes 1 and 2, respectively). The parity effect took account of first, second, third and more lambing (classes 1, 2 and 3, respectively). The effect of litter size class took account of the number of lambs born and suckled (class 0, empty ewes or lambing but without suckling lambs; class 1, ewes lambing and suckling singleton from L to W; class 2 ewes lambing more than singleton and suckling one lamb; class 3, ewes lambing and suckling twins; and class 4, ewes lambing and suckling more than twins). At mating, the effect of litter size class from the previous parity was considered. The first-order interactions between age at first lambing × litter size class and parity × litter size class were tested. An effect was considered significant if *P* < 0.05.

### Phenotypes

First, raw phenotypes were adjusted for significant fixed effects consisting of age at first lambing, parity of the ewe, litter size class, and year of measurement fitting the following linear model:

$$\mathrm{y}=\mathrm{Xb}+\mathrm{e},\kern0.5em \left[\mathrm{Model}\ 1\right]$$

where y is the vector of observations for one of the BR traits; b is the vector of fixed effects; and e is the vector of random residuals with incidence matrix X. The residuals resulting for this model (1) were used as adjusted phenotypes to fit an animal model to estimate individual values using ASREML 3.0 software [39] with the following linear mixed model:

$$\begin{array}{cc}\mathrm y^\ast=\mathrm{Zc}+\mathrm e,&\left[\mathrm{Model}\;2\right]\end{array}$$

where y* is the vector of adjusted phenotypes; c is the vector of random animal effects; and e is the vector of random residuals with incidence matrix Z. c and e were assumed to be normally distributed with means equal to zero and (co) variances Iσ^2^c, Iσ^2^e, respectively. I are identity matrices of appropriate size. Model 2 was fitted with an identity matrix I for random animal effects fitted instead with the pedigree-based relationship matrix, as performed in Mucha et al. [40]. The resulting estimated animal values were used as phenotypes for subsequent genomic analyses instead of estimating breeding values. This was done to take repeated measurements for each individual into account, considering animal effect as a permanent environmental effect and not to use estimated breeding values that give very high false positive rates (i.e. increase type 1 error rates) due to influence of pedigree relationships (contributions of parents and relatives) on an animal’s value [41].

### SNP genotypes and quality control

Sheep were commercially genotyped with Illumina Ovine SNP15K (i.e., 16,560 Single Nucleotide Polymorphisms (SNP); low density (LD)), SNP50K (i.e., 54,241 SNPs, medium density (MD)) or SNP600K (i.e., 606,006 SNPs; high density (HD)) beadchips. Genotypes were established as part of the research projects: “SheepSNPQTL”, “COMPAGNE”, “RomaneIteDomum”, “iSAGE” and “SMARTER”. A total of 1034 phenotyped female animals were genotyped and distributed as follow: 820 ewes were genotyped with the MD beadchip, 167 ewes were genotyped with the LD beadchip, and 47 ewes were genotyped with the HD beadchip. Among the phenotyped and genotyped females, 554 have their dam genotyped (i.e., 389 genotyped dams out of a total of 700 dams) and 965 have their sire genotyped (i.e., 49 genotyped sires out of a total of 60 sires). Dams and sires were genotyped with either MD or HD chips (374 parents and 64 parents, respectively). Concerning ewes genotyped with LD chip, both parents were genotyped.

Individuals with a call rate below 0.95 and with Mendelian inconsistencies were discarded (i.e., five genotyped and phenotyped ewes were removed). The SNPs were removed from further analyses if they were not in Hardy-Weinberg equilibrium, had a minor allele frequency below 1% or had a call rate below 0.98. PLINK software was used to detect incompatible genotypes between sires, dams and offspring [42]. When the total number of incompatible SNPs was more than 2% of all SNPs, ewes were kept in the analyses and the parents in error were replaced by missing values (14 ewes concerned). For ewes genotyped with the HD chip, only SNPs present on the MD chip and LD chip were kept. The ewes genotyped with the LD chip were imputed to the MD chip using Fimpute software, as were LD chip SNPs absent in the MD chip [43]. This resulted in a data set, used for QTL analyses, containing 1034 ewes with BR phenotypes genotyped for 48,593 autosomal SNPs.

### QTL detection method

A genome-wide association study (GWAS) was performed using a univariate linear mixed model (LMM) to account for relatedness and population structure, as implemented in GEMMA v0.94.1 software [44], assessing significance with the Wald test. The statistical model used to test one marker at a time was:

$${\displaystyle \begin{array}{cc}{\mathrm{y}}^{\#}=\mathrm{Wp}+\mathrm{Zu}+\varepsilon, & \left[\mathrm{Model}\ 3\right]\end{array}}$$

where y^#^ is the vector of phenotypes (i.e. solutions from vector c in model 2) for all individuals adjusted for fixed effects and accounted for repeated measures of animals; W is the vector of genotypes at the tested marker; p is the effect of the tested marker; u is a vector of random additive genetic effects distributed according to N(0, Aλτ^− 1^), where λ is the ratio of the additive genetic variance and the residual variance τ^− 1^, A is the additive relationship matrix, and Z is the incidence matrix (identity matrix in this case); ε is a vector of residuals distributed according to N(0, Iτ^− 1^), where I is the identity matrix.

GEMMA software implements the Genome-wide Efficient Mixed Model Association algorithm. The first step of the analyses included the estimation of the relatedness matrix. The resulting metrics were included in the second step (GWAS), allowing for the adjustment for both relatedness and population structure. The significance thresholds at the chromosome-wide level (BONF_chr i_) and genome-wide level (BONF_geno_) were obtained using the Bonferroni method that accounts for multiple testing assuming that the number of independent tests was equal to the number of SNPs analyzed [45]. The formulas to obtain thresholds were the following:

$${\mathrm{BONF}}_{\mathrm{chr}\ \mathrm{i}}=-{\mathit{\log}}_{10}\left(\frac{\alpha }{SNPs\ i}\right)$$

$${\mathrm{BONF}}_{\mathrm{geno}}=-{\mathit{\log}}_{10}\left(\frac{\alpha }{SNPs\ geno}\right)$$

where *SNPs i* is the number of SNPs for chromosome i, and *SNPs geno* is the total number of SNPs at the genome level (i.e., 48,593 autosomal SNPs) and considering *α* = 5%. The resulting genome-wide significance threshold was equal to 5.94. GWAS was performed for all of the 26 autosomal chromosomes. The percentage of variance explained by each SNP was calculated as follows:

$$\%{\sigma}_p^2=100\left(\frac{2p\left(1-p\right){\alpha}^2}{\sigma_p^2}\right)$$

where ${\sigma}_p^2$ is the phenotypic variance of the trait (i.e. total phenotypic variance was obtained from variance components in model 2), *p* is the frequency of the allelic substitution effect of the SNP, and *α* is the estimated allelic substitution effect of the SNP [46]. When a given trait was significantly affected by multiple variants, the reported top SNPs are SNPs that have the highest –log 10 (*P*-value) among the significant SNPs in a 1-Mbp window.

The annotated candidate genes that were closest to the top SNPs were identified using the Ensembl release 104 of the sheep reference genome OAR v3.1 [47]. Position of the top SNP of QTLs and the annotated genes closest to the top SNPs were updated with the reference genome Rambouillet v1.0 (Additional file 2: Table S3).

### LEPR structure and effect of LEPR genotype

Multiple LEPR protein sequence alignment of several mammals was performed using Weblogo software [23, 48]. Protein sequences are available at NCBI (*Mus musculus* NP_666258.2, Ratus norvegicus NP_036728.1, *Homo Sapiens* NP_002294.2, *Sus scrofa* NP_001019758.1, *Bos taurus* NP_001012285.2, *Ovis aries* NP_001009763.1 incomplete sequence) [49]. The complete LEPR protein sequence in sheep (W5PL31, 1165 amino acids) was obtained from the UniProt data base [50] from transcript ENSOART00000011314.1 (Ensembl), and we introduced the proline to serine substitution at position 1019.

Analyses of variance were performed to test the effect of the LEPR genotype (i.e., SNP oar3_OAR1_40,857,869) on BR phenotypes using the MIXED procedure of SAS (version 9.4; SAS Institute Inc., Cary, NC, USA). The dependent variables were phenotypes used in GWAS for BR and BRD at each physiological stage, as described above. The three possible LEPR genotypes were fitted as a fixed explanatory variable and a significance threshold of *p* < 0.05 was selected. The Varcomp procedure of SAS was used to fit the genotype effect as random and to estimate the proportion of variance explained by the genotype. The animal was included as a random effect in both the Mixed and Varcomp models.

The effects of the LEPR genotype on BW, BF and BM were independently analyzed at each physiological stage with a linear mixed model using the mixed procedure of SAS. The LEPR genotype was included as a fixed effect, whereas the animal was treated as a random effect. Appropriate following fixed effects, parity, litter size class, age at first lambing and year of measurement, were also including in mixed models depending on the trait analyzed.

## Supplementary Information

**Additional file 1: Table S1**: Least-square means for body reserves at each physiological stage of ewes according to year. **Table S2**: Least-square means for body reserve dynamics over successive physiological stages of ewes according to year.

**Additional file 2: Table S3**: Position of the top SNP based on the reference genome Rambouillet v1.0 and closest gene associated with body reserves and body reserves dynamics.

## Data Availability

The phenotypes and genotypes data used for this study are available in the Zenodo public repository (https://zenodo.org/record/5729197, DOI: 10.5281/zenodo.5729197) [51].

## Abbreviations

BRD: Body Reserve Dynamics
BR: Body Reserves
BCS: Body Condition Score
BW: Body Weight
BF: Back fat thickness
BM: Back muscle thickness
M: Mating
Pa: Early pregnancy
Pb: Two-thirds pregnancy
L: Lambing
Sa: Early suckling
Sb: End of suckling
W: Weaning
Wp: Post-weaning
CCL28: C-C motif chemokine 28
CDH6: Cadherin-6
DAB2: Disabled homolog 2
DNAJC6: Putative tyrosine-protein phosphatase auxilin
FREM3: FRAS1-related extracellular matrix protein 3
GAB1: GRB2-associated-binding protein 1
HS3ST2: Heparan sulfate glucosamine 3-O-sulfotransferase 2
KCNMA1: Calcium-activated potassium channel subunit alpha-1
LEPR: Leptin Receptor
MIER1: Mesoderm induction early response protein 1
MYO1B: Unconventional myosin-Ib
STAT1: Signal transducer and activator of transcription 1-alpha/beta
NRG4: Pro-neuregulin-4, membrane-bound isoform
NXPE4: NXPE family member 4
OR10W1: Olfactory receptor 10 W1
PCDH8: Protocadherin-8
PGM1: Phosphoglucomutase-1
SORCS3: VPS10 domain-containing receptor SorCS3
SYT1: Synaptotagmin-1
TPD52L1: Tumor protein D53
