# Supplementary material for: Genome-wide analyses reveal a strong association between LEPR gene variants and body fat reserves in ewes
Source: BMC Genomics. 2022 Jun 1;23:412. doi: 10.1186/s12864-022-08636-z (PMC9158286; doi:10.1186/s12864-022-08636-z)
Supplement: Supplementary file 1 — Additional file 1: Table S1: Least-square means for body reserves at each physiological stage of ewes according to year. Table S2: Least-square means for body reserve dynamics over successive physiological stages of ewes according to year. [file 12864_2022_8636_MOESM1_ESM.docx]

**Table S1.** Least-square means for body reserves at each physiological stage of ewes according to year.

| Year |  | BCS-M | BCS-Pa | BCS-Pb | BCS-L | BCS-Sa | BCS-Sb | BCS-W | BCS-Wp |
| --- | --- | --- | --- | --- | --- | --- | --- | --- | --- |
| N Obs. |  | 2069 | 2167 | 2167 | 2162 | 1983 | 1317 | 2084 | 1776 |
| 2006 |  | 2.68 | 3.06 | 2.88 | 2.64 | 2.73 | 2.87 | 2.77 | 2.82 |
| 2007 |  | 2.81 | 3.05 | 2.99 | 2.67 | 2.62 | ND | 2.66 | 2.63 |
| 2008 |  | 2.87 | 2.92 | 2.82 | 2.60 | 2.52 | ND | 2.58 | 2.64 |
| 2009 |  | 2.68 | 2.80 | 2.74 | 2.59 | 2.49 | 2.57 | 2.61 | 2.65 |
| 2010 |  | 2.76 | 2.89 | 3.0 | 2.66 | 2.61 | 2.57 | 2.62 | 2.58 |
| 2011 |  | ND | 2.82 | 2.62 | 2.50 | 2.51 | ND | 2.56 | 2.71 |
| 2012 |  | 2.9 | 3.01 | 2.89 | 2.72 | 2.60 | ND | 2.53 | 2.70 |
| 2013 |  | 2.93 | 2.98 | 2.75 | 2.68 | 2.63 | 2.52 | 2.55 | 2.79 |
| 2014 |  | 2.92 | 2.91 | 2.69 | 2.78 | 2.72 | 2.77 | 2.66 | 2.75 |
| 2015 |  | 2.89 | 2.98 | 2.68 | 2.77 | 2.47 | ND | 2.50 | 2.45 |
| 2016 |  | 2.64 | 2.85 | 2.64 | 2.46 | 2.51 | 2.64 | 2.52 | 2.48 |
| 2017 |  | 2.68 | 2.90 | 2.69 | 2.75 | 2.63 | 2.46 | 2.47 | 2.56 |
| 2018 |  | 2.55 | 2.88 | 2.85 | 2.61 | 2.60 | 2.54 | 2.25 | 2.44 |
| 2019 |  | 2.82 | 2.91 | 2.58 | 2.79 | 2.48 | 2.55 | 2.43 | 2.65 |
| Sign. |  | *** | *** | *** | *** | *** | *** | *** | *** |
|  |  |  |  |  |  |  |  |  |  |

N Obs., number of records for each trait; ND, not determined. M, Mating; Pa, Early pregnancy; Pb, Two-thirds pregnancy; L, Lambing; Sa, Early suckling; Sb, End of suckling; W, Weaning; Wp, Post-weaning period. Sign., the significance probabilities for year effect are provided as : *** P-value < 0.001.

**Table S2.** Least-square means for body reserve dynamics over successive physiological stages of ewes according to year.

| Year | BCS-M:Pa | BCS-Pa:L | BCS-Pa:W | BCS-L:Sa | BCS-W:Wp | BCS-W:M |
| --- | --- | --- | --- | --- | --- | --- |
| N obs. | 2060 | 2153 | 2075 | 1978 | 1730 | 1204 |
| 2006 | 0.38 | -0.41 | -0.29 | 0.08 | 0.06 | 0.10 |
| 2007 | 0.22 | -0.38 | -0.39 | -0.02 | 0.03 | 0.29 |
| 2008 | 0.05 | -0.32 | -0.34 | -0.06 | 0.09 | 0.08 |
| 2009 | 0.12 | -0.22 | -0.19 | -0.10 | 0.05 | 0.16 |
| 2010 | 0.12 | -0.23 | -0.26 | -0.04 | -0.03 | nd |
| 2011 | nd | -0.33 | -0.25 | 0.01 | 0.14 | 0.20 |
| 2012 | 0.11 | -0.29 | -0.47 | -0.13 | 0.15 | 0.31 |
| 2013 | 0.05 | -0.30 | -0.45 | -0.05 | 0.26 | 0.28 |
| 2014 | -0.02 | -0.15 | -0.26 | -0.05 | 0.10 | 0.23 |
| 2015 | 0.08 | -0.22 | -0.48 | -0.28 | -0.04 | 0.09 |
| 2016 | 0.20 | -0.39 | -0.32 | 0.06 | -0.04 | 0.06 |
| 2017 | 0.23 | -0.14 | -0.40 | -0.12 | 0.10 | -0.22 |
| 2018 | 0.35 | -0.26 | -0.62 | -0.01 | 0.22 | 0.45 |
| 2019 | 0.09 | -0.12 | -0.49 | -0.30 | 0.25 | nd |
| Sign. | *** | *** | *** | *** | *** | *** |

N Obs., number of records for each trait; nd, not determined; M:Pa, Mating to Early pregnancy; Pa:L, Early pregnancy to Lambing; Pa:W, Early pregnancy to Weaning; L:Sa, Lambing to Early suckling; W:Wp, Weaning to Post-weaning; W:M, Weaning to Mating. Sign., the significance probabilities for year effect are provided as : *** P-value < 0.001.
