# Supplementary material for: Genome-wide analyses reveal a strong association between LEPR gene variants and body fat reserves in ewes
Source: BMC Genomics. 2022 Jun 1;23:412. doi: 10.1186/s12864-022-08636-z (PMC9158286; doi:10.1186/s12864-022-08636-z)
Supplement: Supplementary file 2 — Additional file 2: Table S3: Position of the top SNP based on the reference genome Rambouillet v1.0 and closest gene associated with body reserves and body reserves dynamics. [file 12864_2022_8636_MOESM2_ESM.docx]

**Table S3.** Position of the top SNP based on the reference genome Rambouillet v1.0 and closest gene associated with body reserves and body reserves dynamics.

| Trait^1^ | Chr | Top SNP^2^ | variant ID | Position (bp) (Texel v3.1) | Position (bp) (Rambouillet v1.0) | Closest gene^3^ |
| --- | --- | --- | --- | --- | --- | --- |
| **BCS-Pb** | **1** | **OAR1_40030112.1** | rs412153758 | **38,809,812** | **41145789** | ***ROR1*** |
| BCS-L | 1 | OAR1_40030112.1 | rs412153758 | 38,809,812 | 41145789 | *ROR1* |
| BCS-Pa | 1 | s30054.1 | rs401019024 | 40,524,081 | 42964458 | *DNAJC6* |
| **BCS-Sa** | **1** | **oar3_OAR1_40821987** | **rs408148486** | **40,821,987** | **43,168,204** | ***LEPR*** |
| **BCS-Pb** | **1** | **oar3_OAR1_40857869** | rs428867159 | **40,857,869** | **43204167** | ***LEPR*** |
| **BCS-L** | **1** | **oar3_OAR1_40890859** | rs409595375 | **40,890,859** | **43318503** | ***LEPR*** |
| **BCS-Pb** | **1** | **OAR1_42228129.1** | rs426448654 | **42,228,129** | **43169418** | ***LEPR*** |
| BCS-Sb | 2 | OAR2_204233702.1 | rs426541865 | 192,716,355 | 206912599 | *MYO1B* |
| BCS-Pa:W | 3 | OAR3_115100887.1 | rs413366284 | 115,100,887 | 14418793 | *TRHDE, TPH2* |
| BCS-Pb | 8 | s48527.1 | rs417582019 | 13,120,875 | 14803144 | *TPD52L1* |
| BCS-Pa | 10 | OAR10_9706403.1 | rs411564223 | 11,281,919 | 13119288 | *PCDH8* |
| BCS-Pa | 15 | OAR15_26097184.1 | rs410315563 | 24,911,875 | 28025654 | *CADM1* |
| BCS-Sa | 15 | OAR15_87285774.1 | rs420712767 | 78,544,042 | 87721474 | *OR10Q1* |
| BCS-Sa | 15 | OAR15_87912118.1 | rs401912882 | 87,912,118 | nd |  |
| **BCS-W:Wp** | **16** | **OAR16_33763548.1** | **nd** | **31,368,878** | **nd** |  |
| BCS-W:Wp | 16 | OAR16_34857607.1 | rs398255861 | 34,857,607 | 33910252 | *GHR* |
| BCS-Pa:L | 16 | OAR16_46544413.1 | rs416016434 | 42,819,279 | 45513470 | *-* |
| BCS-Sa | 17 | s70069.1 | rs429987729 | 14,052,727 | 15744966 | *SMARCA5* |
| BCS-L | 18 | OAR18_31578626.1 | rs419138467 | 30,304,217 | 29280051 | *NRG4* |
| BCS-M:Pa | 22 | OAR22_24239807.1 | rs411931163 | 24,239,807 | 23833552 | *PKD2L1* |
| BCS-Pa:L | 24 | OAR24_22245800.1 | rs426954581 | 20,494,588 | 21139785 | *HS3ST2* |
| BCS-W:Wp | 25 | s68395.1 | rs413132237 | 32,693,219 | 32538101 | *LRMDA* |
| BCS-Pa | 25 | OAR25_39067458.1 | rs430515867 | 37,133,591 | 39233707 | *NRG3* |

^1^ BCS, Body Condition Score; M, Mating; Pa, Early pregnancy; Pb, Two-thirds pregnancy; L, Lambing; Sa, Early suckling; Sb, End of suckling; W, Weaning; Wp, Post-weaning period. ^2^The reported top SNPs are SNPs that have the highest –log10(P-value) among the significant SNPs that are in 1-Mb windows. SNPs for which –log10(P-value) reached the genome-wide significance threshold (> 5.98) are reported in bold; the other reported SNPs reached the chromosome-wide significance threshold. ^3^Annotated protein coding genes closest to the top SNP of the QTL region. Chr, chromosome. nd, not determined.
